# One-Step Synthesis Heterostructured g-C_3_N_4_/TiO_2_ Composite for Rapid Degradation of Pollutants in Utilizing Visible Light

**DOI:** 10.3390/nano8100842

**Published:** 2018-10-16

**Authors:** Hui Liu, Zhi-Guang Zhang, Hong-Wei He, Xiao-Xiong Wang, Jun Zhang, Qian-Qian Zhang, Yan-Fu Tong, Hong-Ling Liu, Seeram Ramakrishna, Shi-Ying Yan, Yun-Ze Long

**Affiliations:** 1 Collaborative Innovation Center for Nanomaterials & Devices, College of Physics, Qingdao University, Qingdao 266071, China; lhqddx@163.com (H.L.); zhangzhiguangphysics@126.com (Z.-G.Z.); wangxiaoxiong69@163.com (X.-X.W.); iamjunzhang@163.com (J.Z.); zhangqianABCABC@126.com (Q.-Q.Z.); 17660945629@163.com (Y.-F.T.); 15666428550@163.com (H.-L.L.); 2 College of Science & Information, Qingdao Agricultural University, Qingdao 266109, China; 3 Industrial Research Institute of Nonwovens & Technical Textiles, College of Textiles and Clothing, Qingdao University, Qingdao, 266071, China; hhwpost@163.com; 4 Center for Nanofibers & Nanotechnology, Faculty of Engineering, National University of Singapore, Singapore; seeram@nus.edu.sg

**Keywords:** electrospinning, g-C_3_N_4_/TiO_2_, heterostructures, visible light, photocatalyst

## Abstract

To meet the urgent need of society for advanced photocatalytic materials, novel visible light driven heterostructured composite was constructed based on graphitic carbon nitride (g-C_3_N_4_) and fibrous TiO_2_. The g-C_3_N_4_/TiO_2_ (CNT) composite was prepared through electrospinning technology and followed calcination process. The state of the g-C_3_N_4_ and fibrous TiO_2_ was tightly coupled. The photocatalytic performance was measured by degrading the Rhodamine B. Compared to commercial TiO_2_ (P25^®^) and electrospun TiO_2_ nanofibers, the photocatalytic performance of CNT composite was higher than them. The formation of CNT heterostructures and the enlarged specific surface area enhanced the photocatalytic performance, suppressing the recombination rate of photogenerated carriers while broadening the absorption range of light spectrum. Our studies have demonstrated that heterostructured CNT composite with an appropriate proportion can rational use of visible light and can significantly promote the photogenerated charges transferred at the contact interface between g-C_3_N_4_ and TiO_2_.

## 1. Introduction

Dye wastewater was a kind of industrial organic pollution with large chroma, complex composition and difficult to be biochemically treated. In addition, it caused serious harm to the environment and physical health. With the rapid development of modern society, using of economic, environmental friendly, efficient and convenient photocatalytic technology to control the environmental pollution has attracted widespread attention [1,2]. Among wide variety of research materials, semiconductor materials for its economical, stable, and harmless qualities commonly used in photocatalytic technology, including metal oxides, non-metal oxides, nitrides, sulfides, phosphides et al. [3,4,5,6,7]. As a promising candidate photocatalyst, titanium dioxide (TiO_2_) due to its chemical stability, non-toxicity, strong oxidation and reduction, controllable morphology and low-cost properties stands out among the various studied transition metal oxide semiconductors over the past decades. It has been proved that anatase phase TiO_2_ was considered the most active phase involving in photocatalytic degradation of pollutions [8]. However, some of the inevitable shortcomings inherent in TiO_2_ limited the practical application in photocatalytic process. Firstly, as an n-type semiconductor, TiO_2_ has a wide band gap (3.2 eV) and absorbs only 4% of the ultraviolet (UV) light in sunlight [9]. Secondly, the high recombination rate of photogenerated carriers in TiO_2_ leads to lower quantum efficiency and thus affects the efficiency of photocatalysis [10]. Therefore, it is urgent to find a new strategy for narrowing the band gap of TiO_2_, prolonging the lifetime of photogenerated carriers, and improving the photocatalytic performance of the photocatalyst. To date, many effective strategies have been developed to improve the shortcomings of TiO_2_ such as precious metal deposition, metal and non-metal ion doping, semiconductor bonding, surface modification, and so on [11,12,13,14,15]. According to the research in recent years, the formation of heterostructure with semiconductor materials is an effective means to avoid the shortcomings inherent in TiO_2_. It can be proved that surfacemodification of TiO_2_ with a narrow band gap semiconductor could generate a Type II heterostructure. Aguirre et al. have recently employed Cu_2_O coated by TiO_2_ for improving photocatalytic stability and performance and provided a z-scheme mechanism of charge transfer. Besides, the Cu_2_O/TiO_2_ nanocomposites were created to avoid the typical limitation problems found in photocatalysis [16]. In addition, various semiconductor materials of narrow band gap combined with TiO_2_ have been successfully synthesized, such as CdS, CuS, MoS_2_, etc. [17,18,19]. This strategy can not only extend the light capture range of TiO_2_ to the visible light region, but also separate the photogenerated carriers at the contract interface between two different band gap materials through matched energy levels coupling, thereby enhancing the performance of photocatalytic.

Among the studied various narrow band gap semiconductor materials, the g-C_3_N_4_ with an indirect band gap of 2.7 eV has attracted extensive attention due to its thermal stability, physicochemical stability, excellent photoelectric transmission property, non-toxic and harmless characteristics [20,21,22]. In addition, semiconductor g-C_3_N_4_ was easily available and can be obtained by direct thermal decomposition of precursor materials, for instance, urea, melamine, cyanamide and dicyandiamide [23,24,25,26]. Nowadays, several techniques have been reported to combine TiO_2_ with g-C_3_N_4_ to form composite. The prepared TiO_2_/g-C_3_N_4_ composite (g-C_3_N_4_ and TiO_2_ with a certain ratio) can not only extend the absorption spectrum range of TiO_2_, but also promote the separation efficiency of photogenerated carriers, thus improve the photocatalytic performance [27,28]. For instance, Lu et al. obtained the C-TiO_2_/g-C_3_N_4_ (CNT) composite by the hydrothermal and calcination methods [29]. According to this method, granular TiO_2_ was obtained, which has a smaller specific surface area, resulting in a smaller contact surface with lamellar g-C_3_N_4_. Han et al. fabricated the g-C_3_N_4_/Titanium(IV) n-butoxide (TNBT)/PVP nanofibers in using the facile electrospinning technology with the addition proportion precursors for g-C_3_N_4_ and TiO_2_ was 1:15, and then the fibers were calcination to obtain the g-C_3_N_4_ NSs hybridized N-doped TiO_2_ nanofibers (GCN/NT NFs) composite [30]. Although the heterostructured CNT composite can be directly obtained after calcination, the doped g-C_3_N_4_ content accounted for a limited proportion of the composite material, and the heterostructures formed by the contact between g-C_3_N_4_ and TiO_2_ had few sites, making it difficult to harvest a better photocatalytic effect. Lu et al. reported a mean for combining the layered g-C_3_N_4_ with the rod-like TiO_2_ by using the hydrothermal method and calcination process. In comparison with TiO_2_ and g-C_3_N_4_, the heterostructured TiO_2_-based nanorods/g-C_3_N_4_ (TNRs/g-C_3_N_4_) performed excellent photocatalytic performance and remarkable optoelectronic characteristics for removing heavy metals and degrading rhodamineB (RhB) in wastewater [31]. However, the means of preparing materials was complicated, which required secondary hydrothermal process. Therefore, a novel one-step synthesis method is needed to prepare the CNT composite with large specific surface area and can combine a large amount of g-C_3_N_4_ to provide more contact sites points.

In the current work, we developed the novel method to prepare CNT composites based on the flexible electrospinning technology [32,33,34,35] and calcination process. TBOT/PVP nanofibers were fabricated by electrospinning method, followed by one-step synthesis a type II heterostructure based on n-type g-C_3_N_4_ and n-type TiO_2_ nanofibers in calcination process. The synthesized porous TiO_2_ nanofibers were tightly wrapped on the surface of the g-C_3_N_4_, and the fibrous TiO_2_ with large specific surface area were contacted with the substrate of the lamellar state that was provided by a large amount of g-C_3_N_4_ to form heterostructure. The heterostructured CNT composite was found available in absorbing the visible light and promoting the separation in photogenerated carriers. Consequently, the CNT composite was promising to be applied in practical environmental protection for removing the organic pollutants. 

## 2. Experimental Section

### 2.1. Materials 

Polyvinyl Pyrrolidone (PVP, M_W_ ≈ 1300000) was purchased from Shanghai Aladdin Bio-Chem Technology Co., Ltd. (Shanghai, China). Tetrabutyl orthotitanate (TBOT, CP, 98.0%), tert-butyl alcohol (TBA, CP, 98.0%), melamine (CP, 98.0%), acetic acid (AR, 99.5%), ethanol (CP, 95.0%), silver nitrate (AgNO_3_, AR, 99.8%) and disodium ethylenediaminetetraacetate (Na-EDTA, AR, 99%) were all bought from Sinopharm Chemical Reagent Limited Company (Beijing, China). Degussa P25 (80% anatase and 20% rutile) was purchased from Evonik Degussa Company (Shanghai, China). All chemical reagents we used as received without further purification.

### 2.2. Preparation of TBOT/PVP Nanofibers Membrane

0.3 g of PVP, 5 g of alcohol and 2 g of acetic acid were weighed into a prepared 50 mL Erlenmeyer flask and mixed well. Then 3 g TBOT was added into the transparent solution and continued for stirring at 30 min until uniform. Later, 5 mL solution was taken into a syringe and placed on an electrospinning device with a voltage of 10 kV. The metal needle was connected to the anode of the high voltage power supply, and the negative pole of the high voltage power supply was connected to the collector. Followed, the prepared TBOT/PVP nanofibers membrane was collected and dried at 60 °C for 12 h.

### 2.3. Preparation of g-C_3_N_4_.

g-C_3_N_4_ was prepared following the method reported in the literature [36]. Ten g of melamine was uniformly dispersed in an alumina crucible and placed into a muffle furnace by annealing at 550 °C for 4 h to obtain the yellow powder.

### 2.4. Fabrication of CNT Composites

The as-prepared white TBOT/PVP nanofibers membrane and g-C_3_N_4_ powder were weighed in various certain ratios and grounded in a mortar for 1 h to acquire the uniform g-C_3_N_4_/TBOT/PVP composites, as listed in Table 1. Subsequently, the mixture was placed into a crucible and then transferred to a muffle furnace by annealing at 600 °C for 2 h. Anneal operation was to remove the PVP present in the nanofibers for leaving bare TiO_2_, meanwhile allowed the fibrous TiO_2_ tightly combined with g-C_3_N_4_. In contrast, the TBOT/PVP nanofibers membrane was also annealed in the muffle furnace under the same conditions to obtain bare TiO_2_ nanofibers.

### 2.5. Characterization

X-ray diffraction (XRD) patterns were recorded by a Rigaku SmartLab X-ray diffractometer (Rigaku, Tokyo, Japan) using Cu-Kα radiation (λ = 1.54178 Å) with an accelerating voltage at 40 kV, a sweep step was 5° in the 2θ range from 10° to 80°. The morphology and microstructure images of the as-synthesized samples were monitored by a JEOL JSM-7800F field emission scanning electron microscope (SEM) (JEOL, Tokyo, Japan). A JEOL JEM-2100F transmission electron microscopy (TEM) (JEOL, Tokyo, Japan) attached with energy dispersive spectroscopy (EDS) was used for observation the as-prepared samples. X-ray photoelectron spectroscopy (XPS) measurement was taken on a Thermo Scientific Escalab 250Xi system (Thermo Scientific, Shanghai, China) with an Al Kα X-ray source to confirm the surface chemical composition of the samples and the valence state of the contained elements. The Brunauer–Emmett–Teller (BET) specific surface areas of the samples were carried out by Quantachrome Autosorb-IQ-MP/XR nitrogen adsorption apparatus (Quantachrome, Shanghai, China). A Hitachi F-4600 fluorescence spectrometer (Hitachi, Tokyo, Japan) was used to measure the photoluminescence (PL) spectra for studying the recombination efficiency of photogenerated carriers with an excitation wavelength of 320 nm. UV-Vis diffuse reflectance spectra of the as-prepared solid composites photocatalysts (the BaSO_4_ powder was used as a reflectance standard) and UV-Vis absorbance spectra of the reaction solution were collected on a PERSEE-T9 UV-Vis spectrophotometer (PERSEE, Beijing, China).

### 2.6. Photocatalytic Performance

The photocatalytic performances of the samples were determined by evaluating the concentration of representative pollutants RhB 5 mg/L in the 50 mL solution after irradiation with an 800 W Xe lamp equipped with the filter at 420 nm (only launched visible light). 50 mg photocatalysts was added to the dye solution and continue to stir for 30 min in dark state before turning on the Xe lamp, ensuring the equilibrium of adsorption desorption is achieved [37,38]. The solution was illuminated with a visible light source went on for 2 h during the entire photocatalytic process. Under the action of flowing cooling water circulation system, after every 15 min, 4 mL of the solution was extracted from quartz tube and centrifuged at 10,000 rpm speed for 10 min to separate the supernatant. The concentration of RhB in solution was detected by the UV-Vis spectrophotometer (Beijing, China) using an absorption wavelength of 540 nm for indicating the photocatalytic performance. In addition, the roles of various active species in the reaction system were also confirmed by the addition of corresponding scavengers.

## 3. Results and Discussion

### 3.1. Synthesis and Application Process

**Figure 1:**
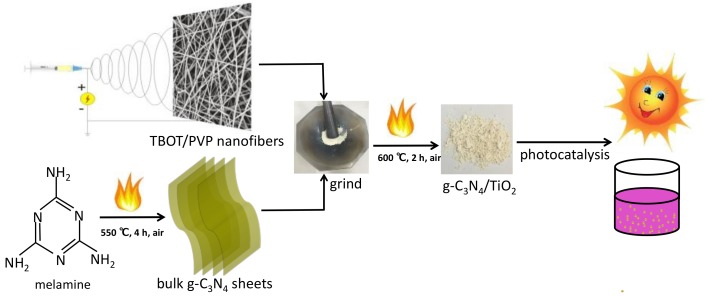
Schematic illustration for the synthesis and visible light photocatalytic application of the CNT composite.

The flow chart of the as-prepared photocatalysts from preparation to application was shown in Figure 1. At the beginning, TBOT/PVP nanofibers were obtained by electrospinning technology [32]. And then the yellow g-C_3_N_4_ was synthesized by calcination the melamine at 550 °C for 4 h, following the method mentioned in the literature [36]. Subsequently, the prepared TBOT/PVP nanofibers were mixed and grinded uniformly with g-C_3_N_4_ powder in a certain proportion and transferred to a muffle furnace for reannealing. Later, the as-prepared various CNT composites were applied to degrade the contaminant under visible light irradiation.

### 3.2. Structure and Morphology Characteristics

**Figure 2:**
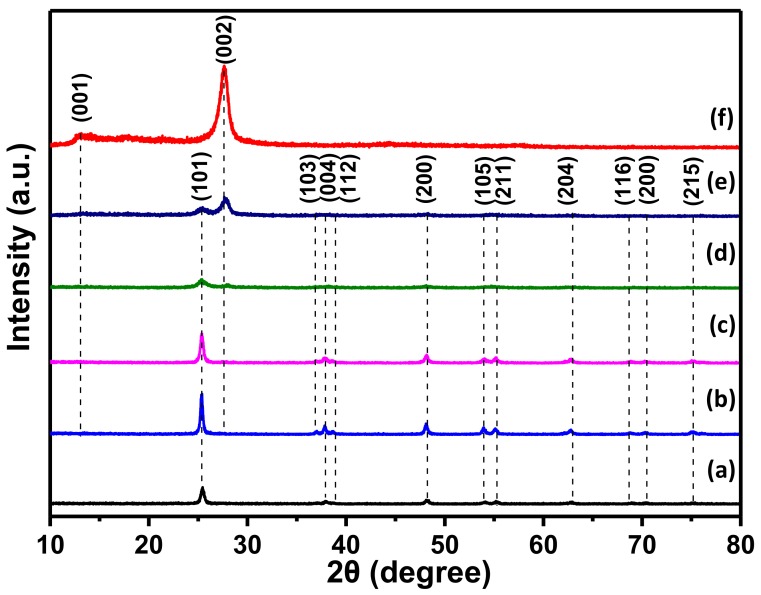
XRD patterns of (**a**) TiO_2_ nanofibers; (**b**) CNT1; (**c**) CNT3; (**d**) CNT5; (**e**) CNT6 and (**f**) g-C_3_N_4_ synthesized at 550 °C.

Figure 2 depicts the XRD patterns of g-C_3_N_4_, TiO_2_ and various CNT composites. As shown in Figure 2 curve a, bare TiO_2_ nanofibers exhibited significant diffraction peaks 2θ at 25.4°, 37.0°, 37.9°, 38.7°, 48.3°, 54.1°, 55.4°, 62.9°, 69.1°, 70.3°, and 75.1°, which were consistent with the (101), (103), (004), (112), (200), (105), (211), (204), (116), (220), and (215) crystal faces of anatase TiO_2_ (JCPDS 21-1272), respectively [39]. In addition, the crystallized peaks 2θ at 13.1° and 27.5° were related to g-C_3_N_4_ phase at (001) and (002) crystal faces (JCPDS 87-1526), respectively [40]. As observed in Figure 2 curves b and c, only bare TiO_2_ was detected and no other peaks appeared in the XRD patterns, owing to the proportion of added g-C_3_N_4_ was less. The diffraction peaks of g-C_3_N_4_ increased markedly with the addition of g-C_3_N_4_ content (Figure 2 curves d and e), indicating a successful compounded of g-C_3_N_4_ to TiO_2_. This phenomenon was also clearly illustrated by SEM images. In addition, the intensity peaks of TiO_2_ become lower for the proportion of g-C_3_N_4_ increases, proving that the content of g-C_3_N_4_ could affect the transformation from TBOT to anatase TiO_2_. Therefore, the results of XRD can inferred that various CNT composites have been successfully synthesized by the process of electrospinning and calcination.

**Figure 3:**
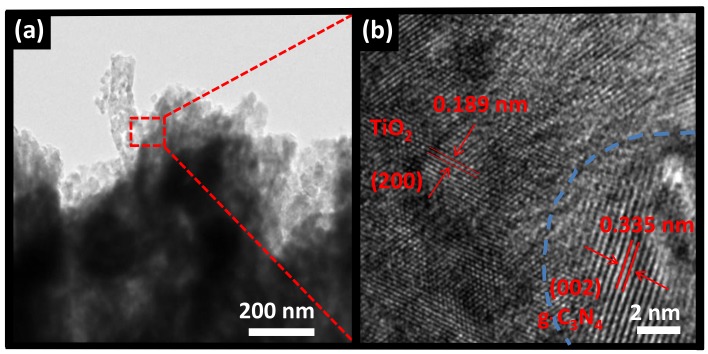
(**a**) TEM and (**b**) HRTEM images of heterostructured CNT6 composite.

The detailed structure of heterostructured CNT6 composite has been further confirmed by TEM and HRTEM, as shown in Figure 3a,b, respectively. In TEM image, it could be clearly seen that the fibrous and bulk substances were apparent as TiO_2_ and g-C_3_N_4_, respectively. It seemed that TiO_2_ was tightly attached the surface of g-C_3_N_4_ so the heterostructure formed, although the thickness of the layer of the latter was uncertain. In addition, the TEM image also investigated that the diameter of the nanofiber was less than 100 nm, which was consistent with the results observed in SEM images (Figure S1). In HRTEM image, the well-ordered lattice spacing of TiO_2_ was measured at 0.189 nm, which was assigned to the exposed (200) plane of anatase TiO_2_ [41]. In addition, the measured lattice spacing of 0.335 nm can be clearly seen, completely ascribed to the (002) plane of g-C_3_N_4_ [42]. Analysis based on SEM, TEM, HRTEM and XPS results for TiO_2_ and g-C_3_N_4_, the formation of CNT heterostructure between g-C_3_N_4_ and TiO_2_ was verified. The close contact interface may contribute to the transfer and separate of photogenerated carriers during the process of light irradiation, which could enhance the performance of photocatalytic. Therefore, the expectation for improving the photocatalytic performance by CNT composite is reasonable.

**Figure 4:**
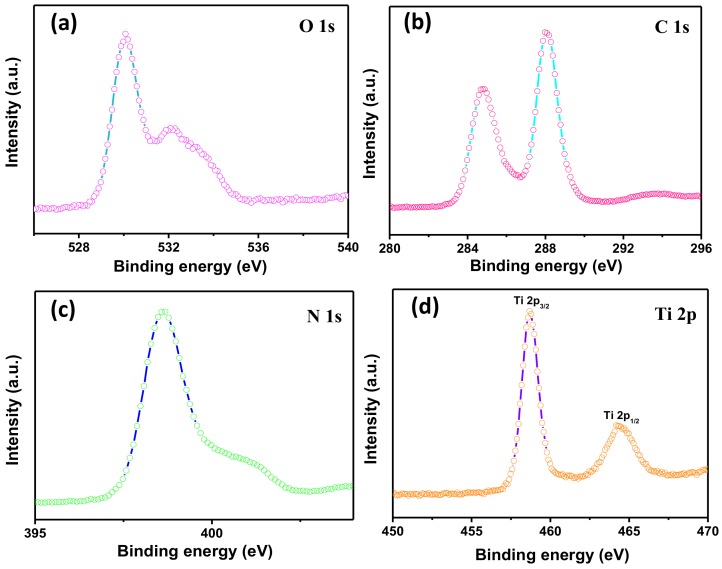
XPS spectra of CNT6: (**a**) O 1s region; (**b**) C 1s region; (**c**) N 1s region and (**d**) Ti 2p region.

The surface chemical composition and elemental states of the as-prepared CNT heterstructured composite were studied using XPS. Figure S2 exhibited the XPS survey spectra included O 1s, Ti 2p, C 1s and N 1s for CNT6 composite. In Figure 4a, O 1s spectrum present two characteristic peaks located at 530.1 eV and 532.2 eV corresponded to Ti–O and C=O, respectively [43]. The spectrum of O 1s indicated that TBOT has been converted to TiO_2_ during the sintering process. The C 1s spectrum in Figure 4b exhibited two major peaks centered at 284.8 eV and 288.1 eV belong to C–C and N–C=N, respectively [44]. Regarding of N 1s spectrum (Figure 4c), the basic units of g-C_3_N_4_ mainly contains three N units, presenting in characteristic peaks to sp^2^ hybridized C=N–C (398.7 eV), tertiary nitrogen N–(C)_3_ (399.6 eV) and C–N–H groups (401.1 eV) [45]. Meanwhile, the peaks of Ti element in Figure 4d distributed at 458.7 eV and 464.5 eV were ascribed to Ti 2p_3/2_ and Ti 2p_1/2_, respectively [46]. The analysis of XPS confirmed the presence of TiO_2_ and g-C_3_N_4_ in the composites. The results further indicate that the g-C_3_N_4_/TiO_2_ heterostructures have been successfully formed, which was matched well with the results obtained by XRD, TEM, UV and PL.

**Figure 5:**
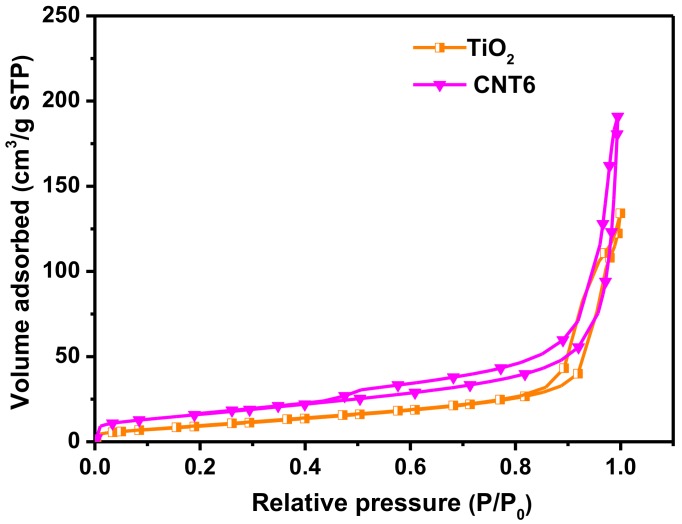
Nitrogen adsorption–desorption isotherms.

Nitrogen adsorption–desorption isotherms analysis was carried out in using BET method to characterize the properties the specific surface area of TiO_2_ nanofibers and CNT6 composite (Figure 5) and the corresponding pore size distribution were recorded by using Barrett–Joyner–Halenda (BJH) method (Figure S3). According to the classification of IUPAC [47], both TiO_2_ nanofibers and CNT6 composite adsorption branches curves exhibit the typical type IV isotherm with H3 hysteresis loops in the relative pressure (P/P_0_) range about 0.5–1.0, reflecting the slit-like mesopores appear in the products. By BET method, the specific surface area and pore volume of CNT6 were calculated to be 58.71 m^2^ g^−1^ and 0.243 cm^3^ g^−1^, respectively, which was much super than bare TiO_2_ nanofibers (34.95 m^2^ g^−1^ and 0.18 cm^3^ g^−1^). The phenomenon might due to the presence of g-C_3_N_4_, which possessing a large specific surface area and have extensive contract with TiO_2_ to inhibit the stack between g-C_3_N_4_ sheets. It could be deduced that heterostructured CNT6 composite was more suitable for meeting the demand as a photocatalyst than bare TiO_2_ nanofibers, owing to the more adsorption reactive contact sites could be provided during the photocatalytic degradation process. Besides, the pore diameter distribution of CNT6 composite (16.53 nm) tends to be smaller compared to TiO_2_ nanofibers (20.58 nm), might due to the CNT6 composite surface was covered by TiO_2_ nanofibers.

### 3.3. Optical Characteristics

**Figure 6:**
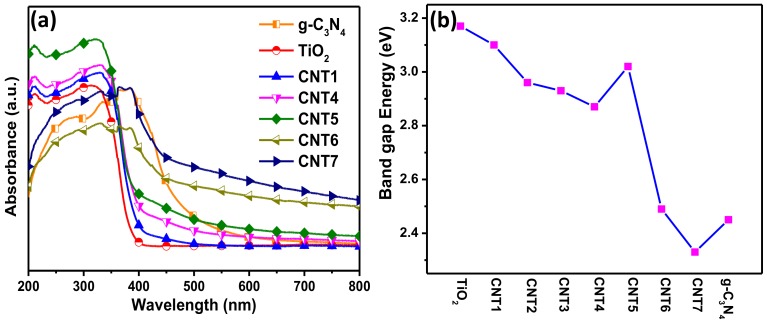
(**a**) UV-vis diffuses reflectance spectra of g-C_3_N_4_, TiO_2_ nanofibers and various CNT composites; (**b**) The curve of band gap energy in various samples.

The optical absorption properties of g-C_3_N_4_, TiO_2_ and various CNT composites were obtained by UV-Vis diffuse reflectance spectra. It can be seen from Figure 6a that all materials have strong absorption in corresponding response light source region. As expect, g-C_3_N_4_ with the intrinsic band gap at 2.7 eV exhibits a significant absorption edge at around 450 nm in visible region [48]. The bare TiO_2_ nanofibers showed the absorption edge at the wavelength of lower than 400 nm in UV region, ascribing to the band gap of 3.2 eV [49]. After compounding the g-C_3_N_4_ with TiO_2_, the CNT composites present evident hybrid adsorption features that the background to capture visible light obviously increased with the increasing of g-C_3_N_4_ content in the composites. Obviously, red shift phenomenon has occurred on composites with the increasing of g-C_3_N_4_ content, performing that the absorption edge moved moderated toward the infrared zone. The situation was also consistent with the color changes of the resulting products from light yellow to black with the increase of g-C_3_N_4_ added amount as shown in Figure S4. The band gap energy range of various CNT composites was estimated at around 2.3 eV–3.1 eV as displayed in Figure 6b. It was calculated by extrapolating the linear region of (hυ·F(R))^1/2^ versus hυ plot to zero F(R), where hυ is the incident photon energy and values of F(R) can be obtained by using the Kubelka−Munk function: F(R) = (1−R)^2^/2R [50,51]. The initial curves of (hυ·F(R))^1/2^ versus hυ were originated from the diffuse reflectance spectra as shown in Figure S5. Thus, it can be concluded that the enhanced ability of CNT composites for capturing the light source could contribute to the improvement of photocatalytic performance.

**Figure 7:**
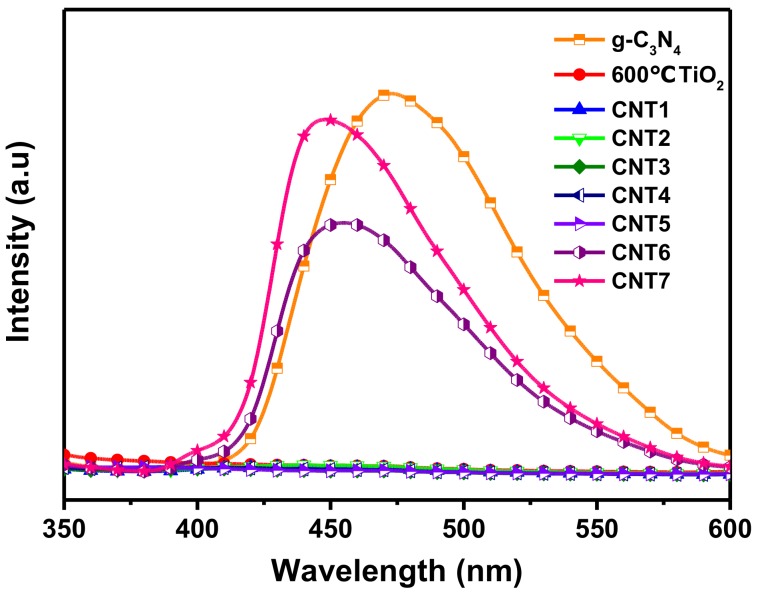
PL spectra of TiO_2_ nanofibers and various CNT composites.

Since the PL emission is derived from the recombination of photogenerated carriers, PL analysis is an effective technique used in photocatalytic process to investigate the transfer, migrate, and separate of electrons and holes. PL spectra of TiO_2_ nanofibers and various CNT composites were recorded under the excitation wavelength of 320 nm (Figure 7). For pure g-C_3_N_4_, one strong peak centered could see at 473.2 nm was ascribed to the band-band PL phenomenon with the light energy bordering on the band gap energy of g-C_3_N_4_ [52]. The PL intensities of all CNT composites were lower than pure g-C_3_N_4_, one reason is that the formation of heterostructures indeed promote the separation of photogenerated carriers at the integrated interface, another reason probably because that g-C_3_N_4_ occupied is less than the single g-C_3_N_4_ in the equivalent amount of the products. Comparing the curves of CNT6, CNT7 and g-C_3_N_4_, it was not difficult to find that the intensity of the emission peak has dropped sharply, which was related to the coupled amount of TiO_2_. Further study the enlarged view of partial curves in Figure 7 as depicted in Figure S6, the PL intensity of CNT1, CNT2, CNT3, CNT4, CNT5 were both decreased than bare TiO_2_, even the PL intensity of CNT3, CNT4, CNT5 were below than the commercial P25^®^. Usually, the PL intensity is inversely to the recombination efficiency of the photogenerated carriers. In other words, the lower the PL intensity, the longer the lifetime of the electrons and holes are, and the better performance performs of the corresponding photocatalyst [53]. Therefore, a suitable ratio of CNT composite is beneficial to improve the photocatalytic performance towards degrading pollutants.

### 3.4. Photocatalytic Performances

**Figure 8:**
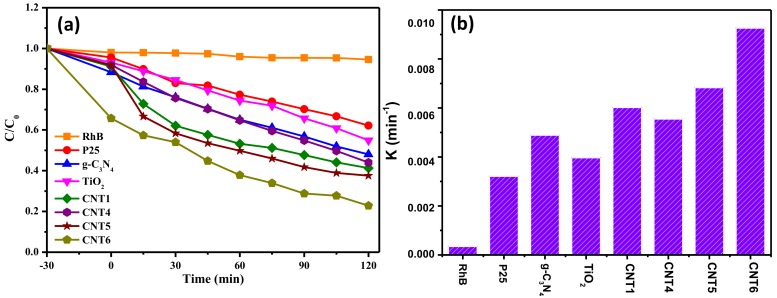
(**a**) Photocatalytic degradation RhB curves under visible light irradiation over different samples; (**b**) reaction rate constant for degradation RhB over different as-prepared photocatalysts.

The photocatlytic performances of the as-prepared samples were evaluated by monitoring the concentration changes in RhB over time under visible light irradiation. In addition, the system was adsorbed in the dark for 30 min to ensure an adsorption-desorption balance achieved. Figure 8a presents the comparison curves of the concentration in RhB degradation by pure g-C_3_N_4_, bare TiO_2_ nanofibers, commercial P25^®^ and partial as-prepared CNT composites within the same time. After dark state adsorption, CNT6 exhibits the best adsorption effect due to the large specific surface area. Figure 8a showed that the concentration changes of RhB can be neglected throughout the photocatalytic process in the absence of photocatalyst, indicating that RhB was relatively stable under visible light irradiation. It can be seen from Figure 8a that the CNT composites reaction system has better photocatalytic performance for degradation of RhB than single material and commercial P25^®^ reaction systems, which was attributed to the effective separation of photogenerated carriers at the intimate contact interface and the enlarged specific surface area. Surprisingly, the wide band gap of TiO_2_ has restricted the range of light absorption wavelength, while it still has good photocatalytic performance in the visible light range. One detailed reason could ascribe that the RhB chromosphere absorbed the visible light and caused the electrons from ground state jumped to the excited state. Subsequently, the excited state electrons rapidly transferred to the conduction band (CB) of TiO_2_, resulting in degradation of RhB [54]. Another reason might owing to the material inherent oxygen vacancy defects and thus altered the range of absorbed light to degradation the RhB. As expect, the as-prepared heterostructured CNT6 showed the optimal photocatalytic performance instead of the as-prepared maximum g-C_3_N_4_ amount loaded in the composite (Figure S7). Hence, it should be noted that the suitable amount of g-C_3_N_4_ bonded in the composite could greatly affect the performance of photocatalysis. The results indicated that the as-prepared product had excellent photocatalytic performance and was suitable as a photocatalyst for harnessing the actual environmental pollution. The photocatalytic degradation of RhB could be expressed as a pseudo-first-order kinetics process, which followed the equation below: [55]

−ln(C/C_0_) = k_app_t

where k_app_ was the reaction rate constant, and C and C_0_ were the concentration of RhB detected at initial t_0_ and t, respectively. Figure 8b displayed the reaction rate constant of degradation RhB over different as-prepared photocatalysts, which was stemmed from Figure S7. According to the equation, the reaction rate constant of CNT6 was calculated as 9.83 × 10^−3^ min^−1^, which was nearly 2.5 times and 3 times higher than that of TiO_2_ nanofibers (4.21 × 10^−3^ min^−1^) and commercial P25^®^ (3.41 × 10^−3^ min^−1^), respectively.

### 3.5. Reaction Mechanisms

**Figure 9:**
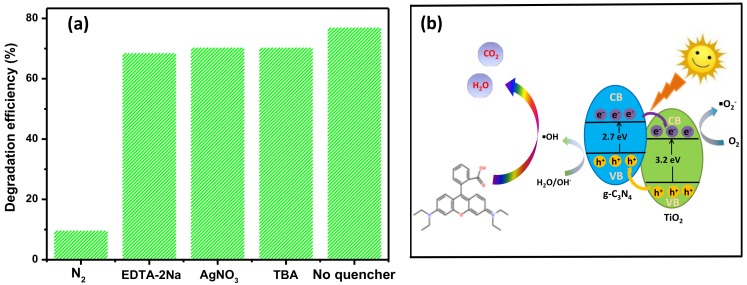
(**a**) Effects on photocatalytic performance by adding different kinds of scavengers; (**b**) Schematic illustration for the separation and recombination process of photogenerated carriers between TiO_2_ and g-C_3_N_4_ under visible light irradiation.

To further study the possible reaction mechanism of the as-prepared photocatalyst, series comparative experiments of radical scavengers were carried out in the reaction system to demonstrate the active species that dominated the photocatalysis process. Added scavengers of N_2_, EDTA-2Na, AgNO_3_ and TBA were correspond for quenching active species of $O_{2}^{-}$, h^+^, e^−^ and OH, respectivity. As can be seen in Figure 9a, the performances of photocatalysis both have the minor changes in the addition of EDTA-2Na, AgNO_3_ and TBA, respectively, meaning that h^+^, e^−^ and OH played a weaker role in the decomposition of RhB. When N_2_ was added into the solution, the remove rate of RhB over CNT6 reduced drastically, suggesting that $O_{2}^{-}$ played the leading role in the photocatalytic process.

Based on the above results, a reasonable photocatalytic mechanism of heterostructured CNT for degradation RhB under visible light irradiation was proposed in detail, as displayed in Figure 9b. Under the irradiation of visible light, the photogenerated electrons (e^−^) of g-C_3_N_4_ and TiO_2_ were both excited from valence band (VB) to CB, leaving the same amount of holes (h^+^) on the VB. Since the conduction edge potential of TiO_2_ was higher than that of g-C_3_N_4_, the electrons of g-C_3_N_4_ on CB were then quickly flowed into the CB of TiO_2_ through the contact interface. Correspondingly, the holes on VB of TiO_2_ were rapidly transferred into the VB of g-C_3_N_4_ because the VB potential of former was more negative than latter. Eventually, photogenerated carriers were separated at the intimate interface, thus prolonging the survival time of electrons and holes. The oxygen radicals ($O_{2}^{-}$) attached to the surface of the photocatalyst were generated from that the electrons have transferred to the CB of TiO_2_ been captured by O_2_ in the solution, possessing strong oxidizing properties on the surface of the photocatalyst ($O_{2}+e^{-}\to O_{2}^{-}$) [56]. Accordingly, the holes moved to the VB of g-C_3_N_4_ having strong oxidative could react with H_2_O or O_2_ to yield hydroxyl radicals $\left(\mathrm{OH}\right)\left(H_{2}O,{\mathrm{OH}}^{-}+h^{+}\to \mathrm{OH}\right)$ [57]. Oxygen radicals and hydroxyl radicals possessed strong oxidizing, can effectively oxidized the organic pollutant RhB in solution transferred into H_2_O and CO_2_. The mechanism of degradation pathway for RhB was proposed. The degradation of RhB mainly via two steps: N-demethylation and cracking of the conjugated structure. After the main steps of chromophore crack, ring opening and mineralization were completed in the photocatalytic process, the dye was converted to smaller organic species. Finally, the products were mineralization with other organic functional groups to form H_2_O and CO_2_ [58,59]. It is worth noting that the content of g-C_3_N_4_ in CNT composites has a significant influence on the separation and recombination efficiency of photogenerated carriers, which has been discussed in PL spectra. An optimal ratio of CNT composite could behave the outstanding photocatalytic performance towards degradation of pollutants under the visible light irradiation.

## 4. Conclusions

In this paper, novel heterostructured g-C_3_N_4_/TiO_2_ (CNT) composites were successfully fabricated by using g-C_3_N_4_ precursor and TBOT/PVP nanofibers via the electrospinning technology [60,61,62,63,64], grinding treatment followed by the calcination process. Under the visible light irradiation, CNT composites exhibited considerable photocatalytic performance on the photocatalytic process for purifying organic contaminant as comparing with commercial TiO_2_ P25^®^ and pure electrospun TiO_2_ nanofibers. An appropriate amount of g-C_3_N_4_ addition can expand the absorption light range of the composites to the visible light region and enlarge the specific surface area of as-prepared materials. The enhanced performance of the photocatalyst was attributed to the formation of heterostructures between g-C_3_N_4_ and TiO_2_ nanofibers, thus the charges on tightly-coupled interface can quickly transferred and separated. Based on the admirable photocatalytic performance of CNT composite in the process of photocatalysis, the as-prepared products can be treated as prospective materials to government the pollutants in environmental.

## Figures and Tables

**Table 1:** Description of various as-prepared g-C_3_N_4_/TiO_2_ (CNT) composite.

Samples	CNT1	CNT2	CNT3	CNT4	CNT5	CNT6	CNT7
g-C_3_N_4_ (g)	0.25	0.3	0.5	1	2	3	4
TBOT/PVP (g)	1	1	1	1	1	1	1

## Supplementary Materials

The following are available online at http://www.mdpi.com/2079-4991/8/10/842/s1, Figure S1: SEM images of (a) g-C_3_N_4_, (b) TiO_2_ nanofibers, (c,d) CNT6 composite, Figure S2: XPS survey spectra of CNT6, Figure S3: The pore size distribution of TiO_2_ nanofibers and CNT6 composite, Figure S4: Optical images of different products: (a) bare TiO_2_ nanofibers, (b) g-C_3_N_4_, (c) CNT2, (d) CNT4, (e) CNT6, and (f) CNT7, Figure S5: The curves of (hν·F(R))^1/2^ versus hν originated from the diffuse reflectance spectra, Figure S6: PL spectra of commercial TiO_2_ P25 and the enlarged view of partial curves in Figure 6, Figure S7: (a) Photocatalytic degradation RhB curves under visible light irradiation over different samples, (b) kinetic curves of degradation RhB over different as-prepared photocatalysts.
