# Supplementary material for: One-Step Synthesis Heterostructured g-C3N4/TiO2 Composite for Rapid Degradation of Pollutants in Utilizing Visible Light
Source: Nanomaterials (Basel). 2018 Oct 16;8(10):842. doi: 10.3390/nano8100842 (PMC6215260; doi:10.3390/nano8100842)
Supplement: Supplementary file 1 [file nanomaterials-08-00842-s001.pdf]

Supporting Information

One-Step Synthesis Heterostructured g-C_3N_4/TiO_2 Composite for Rapid Degradation of Pollutants in Utilizing Visible Light

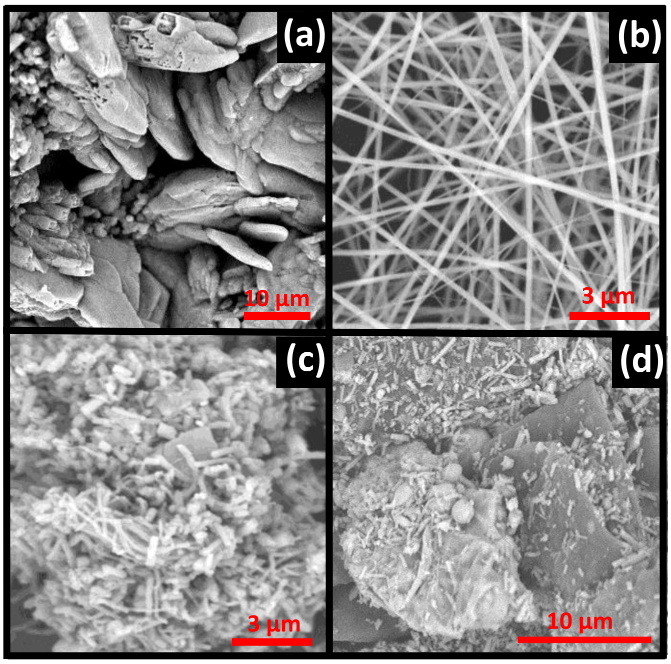

Figure S1. SEM images of (**a**) g-C_3N_4, (**b**) TiO_2 nanofibers, (**c,d**) CNT6 composite.

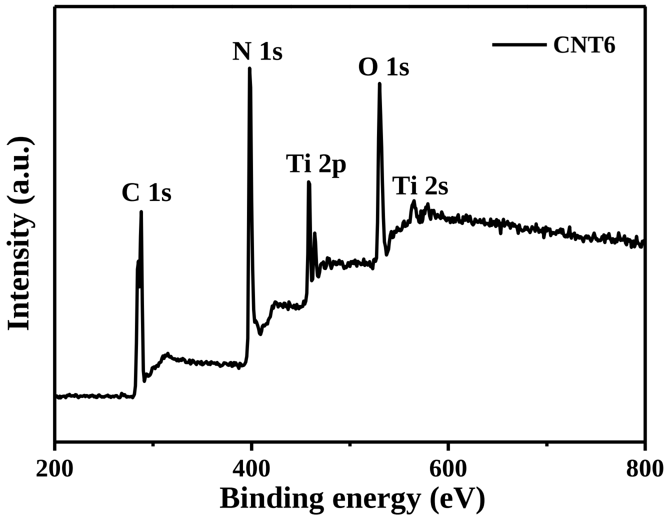

Figure S2. XPS survey spectra of CNT6.

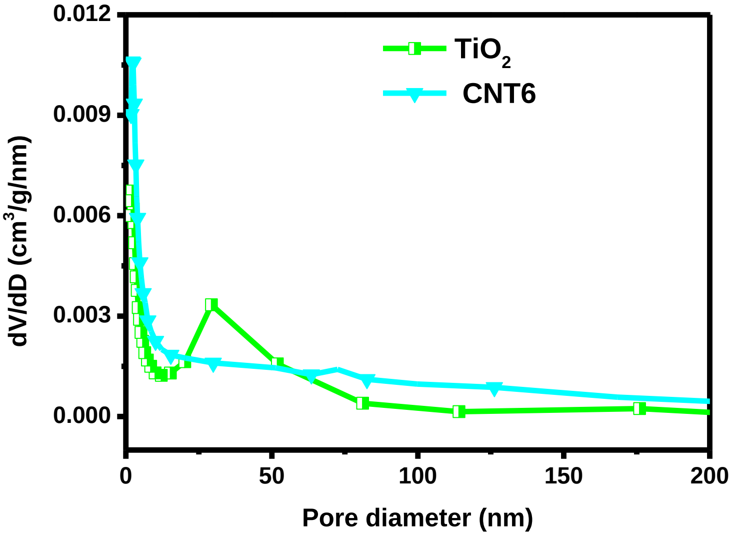

Figure S3. The pore size distribution of TiO_2 nanofibers and CNT6 composite.

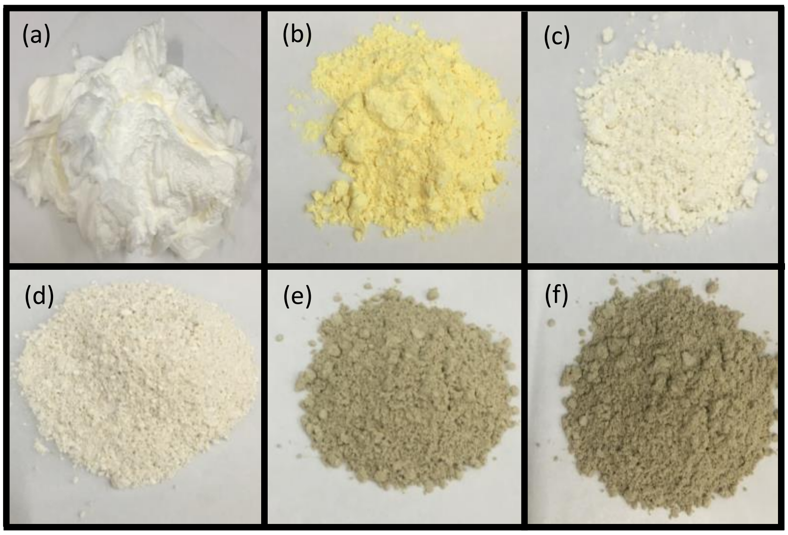

Figure S4. Optical images of different products: (**a**) bare TiO_2 nanofibers, (**b**) g-C_3N_4, (**c**) CNT2, (**d**) CNT4, (**e**) CNT6, and (**f**) CNT7.

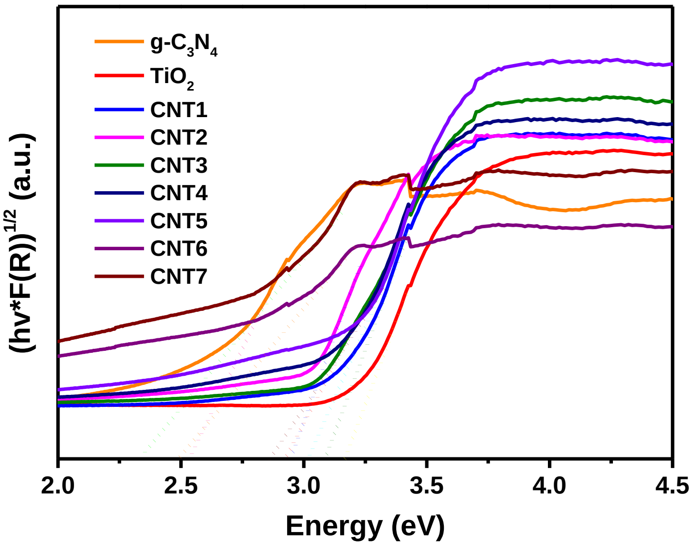

Figure S5. The curves of $(h\nu \cdot F(R))^{1/2}$ versus hυ originated from the diffuse reflectance spectra.

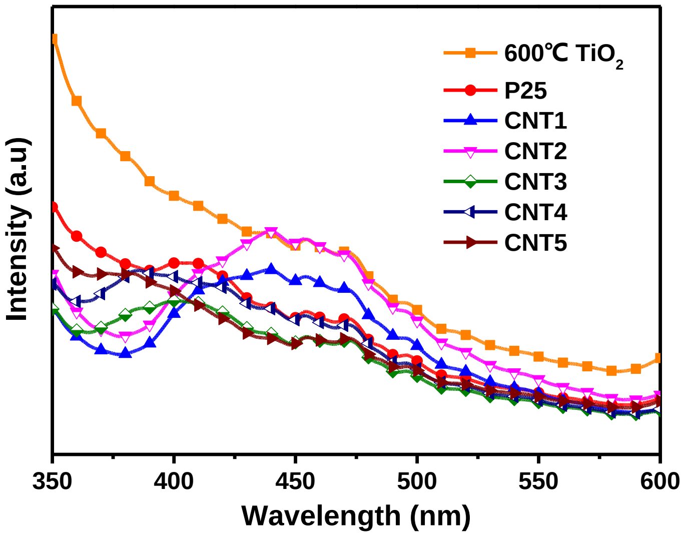

Figure S6. PL spectra of commercial TiO2 P25 and the enlarged view of partial curves in Figure 6.

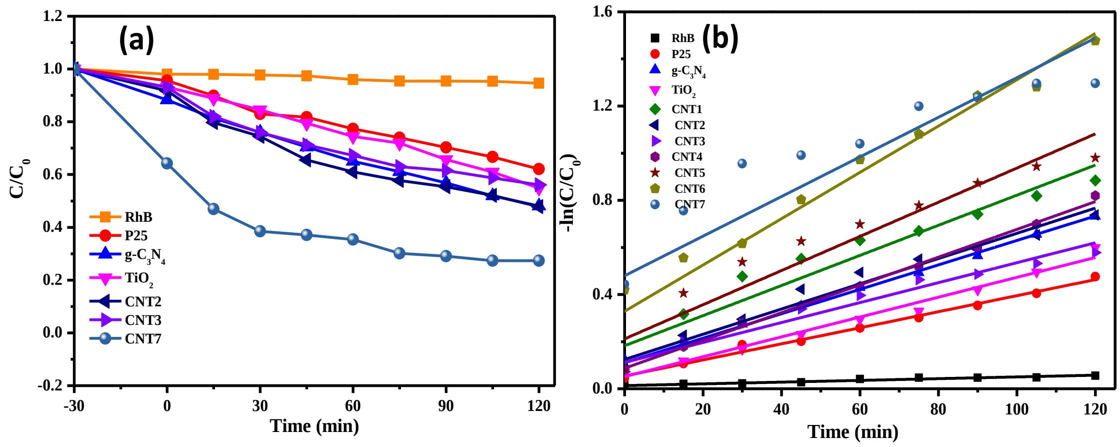

Figure S7. (**a**) Photocatalytic degradation RhB curves under visible light irradiation over different samples, (**b**) kinetic curves of degradation RhB over different as-prepared photocatalysts.
